# Genotyping of *Echinococcus granulosus* Isolates from Human in Khorasan Province, North-Eastern Iran

**Published:** 2019

**Authors:** Fariba BERENJI, Seyed Aliakbar SHAMSIAN, Marziyeh NOURI DALOEE, Seyed Hossein FATTAHI MASOOM, Elham MOGHADDAS

**Affiliations:** 1. Department of Parasitology and Mycology, Faculty of Medicine, Mashhad University of Medical Sciences, Mashhad, Iran; 2. Department of Thoracic Surgery, Cardiothoracic Surgery and Transplant Research Center, Mashhad University of Medical Sciences, Mashhad, Iran

**Keywords:** Hydatid, Human, Strain, Iran, *Echinococcus granulosus*

## Abstract

**Background:**

Human hydatidosis is endemic in northeastern Iran. The present study aimed to investigate molecular diversity of *Echinococcus granulosus* isolates collected from human surgically.

**Methods:**

Sixty human hydatid cysts (58 lung cysts and 2 liver cysts) were collected through surgery from Ghaem and Emam Reza hospitals in Mashhad University of Medical Sciences during 2015–2016. Cysts were characterized using polymerase chain reaction-restriction fragment length polymorphism (PCRRFLP) analysis of the internal transcribed spacer 1 (ITS1) gene and sequencing fragments of the genes coding for mitochondrial cytochrome c oxidase subunit 1 (*cox1*) and NADH dehydrogenase subunit I (*nad1*).

**Results:**

Overall, 55 out of 60 *Echinococcus granulosus* cysts (91.6%) were determined as the G1 strain, 4 cases (6.6%) were determined as the G6 strain and 1 sample was not identified.

**Conclusion:**

Although sheep strain (G1) is dominated in human patients in Great Khorasan, the prevention of camel-dog cycle should pay attention in this region.

## Introduction

*Echinococcus granulosus* settle in small intestine of carnivores as definitive host. Almost of herbivores e.g., human, sheep, cattle, buffalo, goat, camel, horse, pig and many of wild ungulates are intermediate hosts (1). In intermediate host, hydatid cyst was created after ingestion egg of *E. granulosus* via contaminated food, vegetable or water. Direct contact with dogs is the most common route of infection transmission. Humans are accidental host of parasitic hydatid disease and it may occur in each body organs (2).

Genetic variability and morphological variations exit within *E. granulosus* that caused G1 to G10 strains (3). Most of the genotype of human cystic echinococcosis in the worldwide is G1 strain (88.44%), after that the genotype G6 (7.34%). G5, G8 and G10 genotypes were recorded rarely from human and no case from G4 genotype have been identified in human up to now (4). Detection of these strains is important for the epidemiology, control, prevention programs, vaccine designs and drug production.

G1, G3 and G6 strain were reported from Iranian patients (5). Nucleic acid sequencing is the gold standard for determining the genotypes of *E. granulosus* (6). Hydatidosis is endemic in Iran (1% of all surgical admissions). Incidence of human hydatid disease in Khorasan area is as high as 4.45 in 100,000 (7, 8). The average number of operated cysts per year was 134.2 (8). Cyst strains were studied in camels, sheep, goats, and cattle in northeast of Iran without any survey on human hydatid cysts (9, 10). Even alveolar echinococcosis in Monkey (*Ateles geoffroyi*) and small mammals were reported in this area (11, 12).

The aim of this study was to strains identification of *E. granulosus* in human in Great Khorasan Province, north-eastern Iran.

## Materials and Methods

### Sample collection

Sixty pieces of germinal layer of cyst including 58 lung cysts and 2 liver cysts were collected from Ghaem and Emam Reza hospitals in Mashhad University of Medical Sciences, Iran in 2015–2016. All patients lived in Mashhad or small cities that located in Great Khorasan including North Khorasan, South Khorasan and Khorasan Razavi.

### DNA extraction

Germinal layer of every cyst was rinsed several times with sterilized distilled water prior to DNA extraction. Total genomic DNA (gDNA) was extracted from each cyst as manufacturer’s instructions (MBST, Tehran, Iran). About 5% Mm of cyst wall lysed in 180 μl lysis buffers, the proteins were degraded with 20 μl proteinase K for 10 min at 55 °C. After adding 360 μl of binding buffer and incubation for 10 min at 70 °C, 270μl of ethanol (100%) were added to the solution. After vortexing, the complete volume was transferred into the MBST column. The MBST column was first centrifuged and then washed twice with 500 μl of washing buffer. Finally, DNA was eluted from the carrier with 50 ml elution buffer. The concentration of DNA was determined by nanodrop and the samples were stored at −20 °C.

### PCR

#### ITS1 Gene

The forward and reverse primers employed in this study for ITS1 were: EgF (forward), 5′-CAGAGCACTTTTGTATGCA-3′); EgR (reverse), (5′-ATGGTTGTTATCGCTG CGA-3′) (9), The following program was used: 5 min incubation at 95 °C to denature double-stranded DNA, 35 cycles of 45 sec at 94 °C (denaturing step), 45 sec at 50 °C (annealing step) and 45 sec at 72 °C (extension step). Finally, PCR was completed with an additional extension step for 10 min.

### PCR-RFLP of ITS1

For PCR-RFLP, the amplified products (20 μl) were digested with five units of the restriction endonuclease Bsh1236I (5U, Fermentas) in a final volume of 25μl for eight hours in 37 °C (9). This enzyme identifies sequences of CG/CG. Restriction fragments were visualised by gel electrophoreses through 4% ethidium bromide agarose gel.

### Cox1 and Nad1 Gene Sequencing

Randomly 10 samples of G1 and 1 sample of G6 pattern of RFLP-PCR amplified by PCR method. Two primers, JB3 (forward), 5′-TTT TTT GGG CAT CCT GAG GTT TAT -3′ and JB4 (reverse), 5′-TAA AGA AAG AAC ATA ATG AAA ATG -3′ were used to amplify a 450 bp fragment of *cox1* gene under the following conditions: initial denaturation 94°C for 5 min, followed by 35 cycles of denaturation at 94 °C for 45 sec, annealing at 50 °C for 45 sec and elongation for 45 sec at 72 °C. Final extension was performed at 72 °C for 7 min (13). Also two primers, MS1 (forward): 5′-CGTAGGTATGTT GGTTTGTTTGGT-3′) and MS2 (reverse): 5′-CCATAATCAAATGGCGTACGAT-3′ were used to amplify a 400 bp with 5 min initial denaturation at 94 °C, and 30 cycles of 30 sec denaturation at 94 °C, 45 sec annealing at 50 °C and 30 sec elongation at 72 °C. Final extension was performed at 72 °C for 5 min (14). *Nad1* and *cox1* genes after purification were sent for sequencing (Macrogen Company, South Korea). The sequences were compared with those previously published in the Gen-Bank database using the BLAST system (http://www.ncbi.nlm.nih.gov/blast/Blast.cgi).

### Ethics

The study adhered to the tenets of the Declaration of Helsinki and was approved by the Ethics Committee at Mashhad University of Medical Sciences (Ethical code: IR.MUMS.fm.REC.1394.443).

## Results

**Fig. 1:**
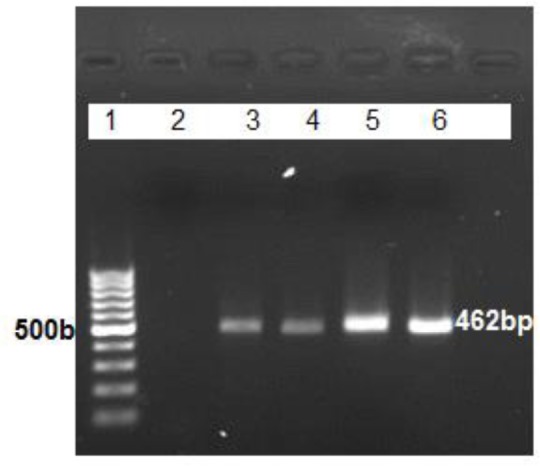
Agarose gel electrophoresis of ITS1-PCR (462 bp) products of *E. granulosus* isolates from human: Lanes 1: ladder 100bp, Lanes 2: negative control, Lane 3, 4, 5: ITS1, Lane 6: positive control

**Fig. 2:**
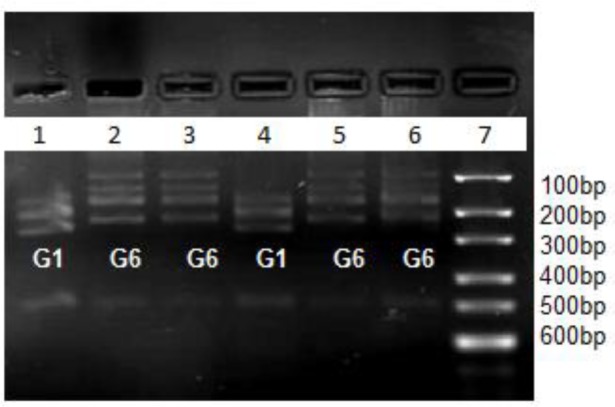
Digestion pattern of PCR products from rDNA-ITS1 fragment with restriction enzyme Bsh1236I: lane 7: marker with 100bp molecular weight, Lane 4, 1: G1 genotype, Lane 2, 3, 5, 6: G6 genotype

We detected fifty-five G1 (sheep strain) and four G6 (camel strain) according to RFLPPCR of ITS1 following were confirmed by sequencing of *cox1* and *nad1* genes. All *E. canadensis* were isolated from lung. One unknown strain did not get satisfied results in RFLP-PCR as same in sequencing result. PCR product of ITS1 gene showed approximately 462 bp in length (Fig. 1). As we expected the restriction enzyme Bsh1236I made three and four bands for G1 and G6 genotype respectively (Fig. 2).

**Fig. 3:**
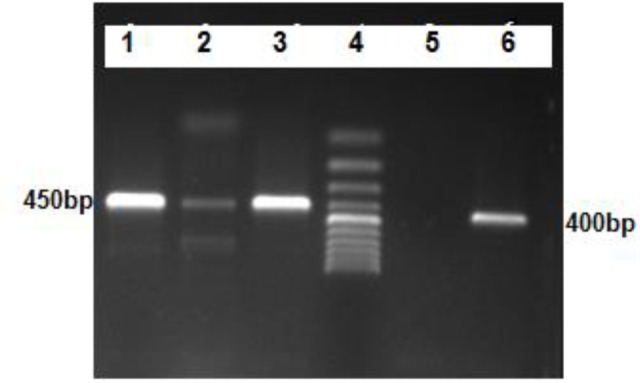
Agarose gel electrophoresis of *cox1*-PCR (450 bp) and nad1 (400 bp) products of *E. granulosus* isolates from human: Lanes 1, 2, 3 *cox1* gene, Lane 4: ladder 100bp, lane 5: negative control and lane 6: *nad1* gene

The partial nucleotide of *cox1* (450bp) and *nad1* (400bp) genes from 10 isolates were amplified by PCR method (Fig. 3). Sequencing of *cox1* and nad1 genes confirmed the RFLP-PCR results. *Cox1* and *nad1* Genes of G6 strain were 100% homology with accession number of Kp751426 and Kp751432 respectively in GenBank. *Cox1* Gene of G1 strain was 99% homology with accession number of MG 322623 in GenBank and showed in comparison with G1 in GenBank a transition of T to A at position 481, A to G at position 650. Moreover, *nad1* Gene of G1 strain was 99% homology with accession number of MG 322623 in GenBank in comparison with G1 in GenBank, a transition of G to A at position 292, T to A at position 418.

### Phylogenetic analysis

**Fig. 4:**
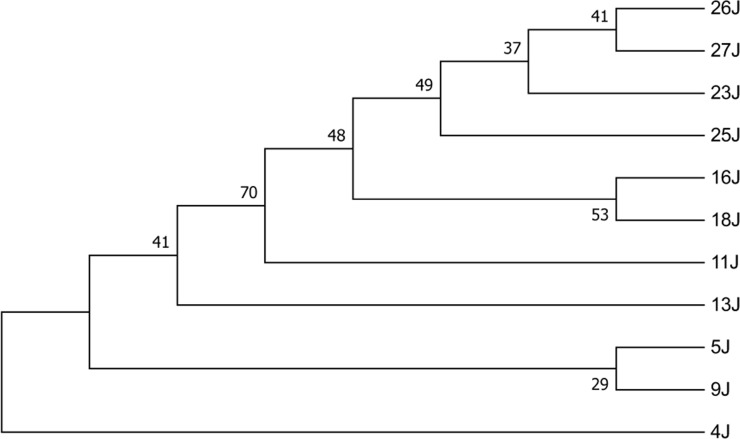
The evolutionary history was inferred by using the Maximum Likelihood method based on the General Time Reversible model. The tree with the highest log likelihood (−629.50) is shown *cox1* sequences of *Echinococcus canadensis* G6 genotype (4J) and G1 genotype (9J, 11J, 13J, 5J, 16J, 18 J, 23J, 25J, 26J, 27J)

**Fig. 5:**
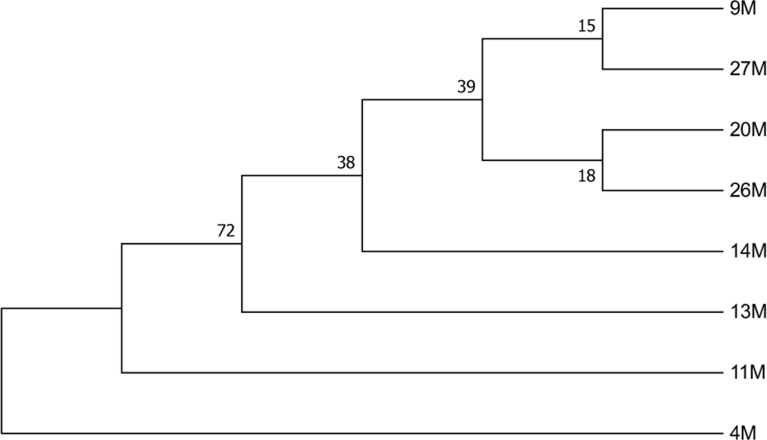
The evolutionary history was inferred by using the Maximum Likelihood method based on the General Time Reversible model. The tree with the highest log likelihood (−814.94) is shown nad1 sequences of *Echinococcus canadensis* G6 genotype (4M) and G1 genotype (9M, 11M, 13M, 14M, 20M, 26M, 27M). There were a total of 319 positions in the final dataset. Evolutionary analyses were conducted in MEGA7

Phylogenetic trees were generated by using *cox1* and *nad1* sequencing (Figs. 4 and 5). Alignment was performed using ClustalW and the aligned sequences manually refined in BioEdi software (version 7.2.5) maximum likelihood (ML) trees were inferred by MEGA 7 software. Nodal support was assessed by bootstrapping with 1000 replicates.

## Discussion

North-east of Iran is hyperendemic for hydatidosis and also alveolar echinococcosis in human and animals (8, 15). Another study has published one thousand and twenty of pulmonary hydatid cyst in different hospitals of Mashhad (capital city of Khorasan Razavi) from 1981 to 2008 (16). During 9 years 1342 human hydatidosis were operated only in three hospitals in this area (8). Hydatid cysts from 70 cattle, 50 sheep and 24 goats were shown G1 genotype in Khorasan Province (10).

In present study dominant strain was G1, followed by G6 which is in accordance with almost other reports in different provinces in Iran (Table 1).

**Table 1:** Published information concerning *Echinococcus granulosus* isolated from humans in different regions of Iran

***Number of human cases***	***Area***	***Strains***	***Method***	***Ref***
4	Tehran	Sensu stricto (G1–G3)	Sequencing of *cox1 and nad1* genes PCR-RFLP	(21)
23	Isfahan	Sensu stricto (G1–G3)	Sequencing of *cox1* and *nad1* genes	(14)
55	Azerbaijan Province	G1	PCR-RFLP (rdna-ITS1)	(22)
4	Ilam Province	Sensu stricto (G1–G3)	PCR-RFLP (rdna-ITS1)	(23)
29	Tehran	G1, G3 and G6	Sequencing of *cox1* and *nad1* genes	(24)
30	Golestan province	G1	PCR-RFLP (rdna-ITS1)	(25)
5	Khuzestan province	G1	PCR-RFLP (rdna-ITS1)	(26)
11	Ardabil Province	G1, G3	Sequencing of cox1 and nad1 genes	(27)
31	Isfahan province	G6, G1	PCR-RFLP (rdna-ITS1)	(28)
12	Different locations of Iran	Sensu stricto (G1–G3)	PCR-RFLP ITS1	(29)
30	Isfahan Province	Sensu stricto (G1–G3)	PCR-RFLP ITS1	(30)
1	Kerman Province	G6	Sequencing of *cox1* and *nad1* genes	(31)
17	Fars Province	8 (G1) and 6 (G6)	Sequencing *nad1* gene	(32)

In the present study 4/60 (6.6%) of all genotype were G6 genotype (camel strain) in human patients. Great Khorasan is neighbour of Afghanistan and annually 4500 of dromedary camels were slaughtered in industrial abattoirs. Interestingly, G6 strain was reported from human in Afghanistan (17). Liver and lung camel hydatidosis were recorded 11.1% and 13.2%, respectively in northeastern Iran (18). Moreover, prevalence of canine *Echinococcosis* in this region was 22% and it was reported to 10,000 worms in one dog intestine (19). This condition can make potential danger of transmission of camel strain to human in northeastern Iran. G6 was responsible for human hydatidosis 9.1% and 40.8% from central and southeast of Iran, respectively (20).

Almost the studies conducted on genotyping of *E. granulosus* in Iran including *cox1* and *nad1* sequencing with or without PCR-RFLP of rDNA-ITS1. By using these techniques G1, G3 and G6 obtained in human and domestic animals in whole the country (Table 1).

Because PCR-RFLP is not capable to differentiate G1–G3 from each other, we did gene sequencing *nad1* and *cox1*genes. We did not find any G3 strain in human cases.

## Conclusion

G1 (sheep strain) is the dominant genotype that involved human hydatidosis in northeastern Iran. In additional G6 (camel strain) observed in this district or the first time in human hydatidosis.
